# IDO Inhibition Facilitates Antitumor Immunity of Vγ9Vδ2 T Cells in Triple-Negative Breast Cancer

**DOI:** 10.3389/fonc.2021.679517

**Published:** 2021-07-22

**Authors:** Peng Li, Ruan Wu, Ke Li, Wenhui Yuan, Chuqian Zeng, Yuting Zhang, Xiao Wang, Xinhai Zhu, Jianjun Zhou, Ping Li, Yunfei Gao

**Affiliations:** ^1^ Zhuhai Precision Medical Center, Zhuhai People’s Hospital (Zhuhai Hospital Affiliated with Jinan University), Jinan University, Zhuhai, China; ^2^ The Biomedical Translational Research Institute, Faculty of Medical Science, Jinan University, Guangzhou, China; ^3^ Department of Infectious Disease, Guangdong Second Provincial General Hospital, Guangzhou, China; ^4^ The First Affiliated Hospital, Jinan University, Guangzhou, China; ^5^ Department of Oncology, First Affiliated Hospital, Jinan University, Guangzhou, China; ^6^ Research Center for Translational Medicine, Cancer Stem Cell Institute, East Hospital, Tongji University School of Medicine, Shanghai, China; ^7^ Department of Endocrinology, Guangdong Second Provincial General Hospital, Guangzhou, China

**Keywords:** IDO, γδT cell, 1-Methyl-L-tryptophan, perforin, anti-tumor immunity

## Abstract

Triple-negative breast cancer (TNBC) escape from immune-mediated destruction was associated with immunosuppressive responses that dampened the activation of tumor-infiltrating CD8 and γδ T cells. TNBC had a higher level of programmed cell death 1-ligand 1 (PD-L1) and indoleamine 2,3-dioxygenase (IDO), compared with other breast cancer subtypes. But, clinical studies have revealed that the response rate of PD-1/PD-L1 antibody for TNBC treatment was relatively low. However, the antitumor responses of human Vγ9Vδ2 T cells or IDO inhibitor in TNBC treatment are unknown. In this study, we found that IDO1 and PD-L1 were highly expressed in TNBC patients. Analysis of the clinical samples demonstrated that Vγ9Vδ2 T cells became exhausted in triple-negative breast cancer patients. And Vγ9Vδ2 T cells combined with αPD-L1 could not further enhance their antitumor responses *in vitro* and *in vivo*. However, Vγ9Vδ2 T cells combined with IDO1 inhibitor 1-Methyl-L-tryptophan (1-MT) or Lindrostat showed substantial inhibitory effects on MDA-MB-231 tumor cells. Finally, we found that IDO1 inhibitor promoted T cell’s cytotoxicity by enhancing perforin production. These results converged to suggest the potential application of Vγ9Vδ2 T cells treated with IDO1 inhibitor for TNBC therapy.

## Introduction

Triple-negative breast cancer (TNBC; estrogen receptor α, progesterone receptor and HER2 (ERBB2)-negative) is the subtype of breast cancers with poorest prognosis due to lack of targeted therapies (1, 2). Immune checkpoint inhibitors (ICIs), such as programmed cell death-1 (PD-1), programmed cell death 1-ligand 1 (PD-L1), and cytotoxic T lymphocyte-associated antigen-4 (CTLA-4), are considered as promising immunotherapeutic strategies for TNBC patients (3); however, the first-in-human treatment of targeting the PD-1/PD-L1 axis for TNBC in clinical trials showed benefits for only a minority of patients (4). Previous findings showed that breast cancer patients with higher levels of infiltrating immune cells had more favorable prognoses, specifically with a higher ratio of tumor-infiltrating cytotoxic CD8 T cells (TILs) to immunosuppressive FoxP3^+^ T regulatory cells (T reg) (5). However, a study on breast cancer demonstrated that tumor-infiltrating γδ T lymphocytes are correlated with a poor prognosis in human breast cancer patients (ER^+^, Her2^+^ positive) (6). Another study showed that γδ T cells infiltrated into various tumors, including TNBCs (7–9), and they appeared to be prognostically beneficial (10). A possible explanation for this contradictory phenomenon was that accumulation of *γ*δ TILs in different breast cancer subtypes might be differently involved in microenvironment and resulted in different outcomes, which were mediated by unknown mechanisms.

The cytosolic enzyme indoleamine 2, 3-dioxygenase (IDO) has been regarded as a potential contributor in breast cancer progression (11). Indoleamine 2, 3-dioxygenase is encoded by the *IDO1* gene and is an intracellular enzyme that participates in the rate-limiting step of the catabolism of L-tryptophan (Trp), an important regulator of amino acid metabolism (12). These enzymes catalyze the oxidation of Trp to N-formyl l-kynurenine (Kyn), which is rapidly converted by formamidases to Kyn (13); however, elevated concentrations of Kyn and high plasma Kyn/Trp ratios frequently occur in patients with advanced-stage cancers and are correlated with poor prognoses (14, 15). IDO expression in the tumor environment (TME) has been linked to the induction of multiple tolerogenic immune phenotypes, including the inhibition of effector T cell activation, enhanced infiltration of myeloid-derived suppressor cells (MDSCs), B cell dysfunction and promotion of tumor angiogenesis (16, 17). Triple-negative breast cancer cells also express IDO in the presence of inflammation and T-cell infiltration (18). Inhibiting of IDO activity could be used to restore tumor immunity in humans, by relieving IDO-mediated immune suppression of MSCs in the TME as well as in tumor cells themselves (19). These inhibitory effects might converge to induce cytotoxic T cell’s exhaustion and dampen antitumor immunity. Tumor cells adopted many approaches to suppress the antitumor immunity mediated by cytotoxic T cells; these approaches included inducing the expression of T-cell immunoglobulin and mucin-domain containing-3 (Tim-3), T cell immunoreceptor with Ig and ITIM domains (TIGIT), CTLA-4 and reducing CD28 expression on T cells in TME (20–22).

1-Methyl-L-tryptophan (1-MT) is an investigational small molecule inhibitor of the IDO enzyme (23). In preclinical report, the combination treatment of 1-MT and anti-PD-L1 could more effectively activate CD8^+^ T cells and inhibit tumor growth than any single one of them (24). One study demonstrated that the combination of navoximod (IDO inhibitor) and atezolizumab (anti-PD-L1) displayed acceptable safety, and tolerability for patients with advanced cancer (25). However, this work showed that there was no clear evidence for benefit of adding navoximod to atezolizumab. Tumor infiltrating CD8^+^ T lymphocytes are considered as an independent prognostic factor associated with improved patient survival in basal-like breast cancers, but not in non-basal triple-negative breast cancers, nor in other intrinsic molecular subtypes (26). Recent study suggested that γδ TILs could be active against breast cancer and other types of tumors (27). Due to their HLA-independent mode of target cell recognition, γδ T cells have recently attracted substantial interest as potential effector cells in cell-based cancer immunotherapy (28, 29). Therefore, an adoptive γδ T cell transfer therapy may be one of the most powerful treatments for patients with malignant tumors.

γδ T cells provide the early source of interferon-γ (IFN-γ) in tumor immunity and have potent cytotoxic property (30). Among the immune cells, γδ T cells (Vγ9Vδ2) have emerged as a newly discovered prospective candidate for solid tumor therapy in clinical trials (31–33). These cells recognize antigens in an MHC-independent manner (non-MHC restriction) *via* surface receptor NKG2D; clinical studies used allogeneic γδ T cells from healthy donors, since γδ T cells from the blood of tumor patients are sometimes difficult to expand *in vitro*. Clinical trial indicated that γδ T-cell transfer was safe and well tolerated (32). Currently, most tumor immune therapy strategies, such as chimeric antigen receptor T cell therapy (CAR-T), were mainly based on αβ T cells (CD4, CD8 T) and had been applied in treating refractory pre-B cell acute lymphoblastic leukemia and diffuse large B cell lymphoma (34, 35). CAR-T cell therapy could induce rapid and durable clinical responses, and sometimes was associated with unique acute toxicities, which could be severe or even fatal to patients (36). ICIs blockade improve the efficacy of current immunotherapies, particularly CAR-T cells, which had shown limited success in treating solid tumors (37, 38). Mouse model data also suggested that fludarabine and cyclophosphamide, frequently administered before CD19-CART infusion to improve their antitumor activity, down-regulated IDO expression in B-cell malignancies (39). However, if combination of human cytotoxic γδ T cells and IDO inhibitor could mediate strong antitumor immunity in TNBC is barely known.

Here, we showed that *IDO1* and *PD-L1* were expressed in TNBC patients, and Vγ9Vδ2 T cells became exhausted. The IDO enzyme inhibitor 1-MT or Lindrostat enhanced the antitumor efficacy of Vγ9Vδ2 T cells for the treatment in MDA-MB-231 tumor model. However, the anti-PD-L1 antibody combined with Vγ9Vδ2 T cells showed no such kind of effects. 1-MT promoted T cell’s cytotoxicity by enhancing perforin production. These results converged to suggest a promotive effect of 1-MT in facilitating the cytotoxic activity of Vγ9Vδ2 T cells in TNBC treatment.

## Materials and Methods

### Ethics Statement

All experiment protocols, including breast tissues or isolation of PBMC from peripheral blood samples of triple negative breast cancer and healthy volunteers, were approved by the Institutional Review Board of Jinan University, Guangzhou, P. R. China (approval number, KY-2020-011).

### Cell Culture and Reagents

Human breast cancer cells (MCF-7 and MDA-MB-231) were obtained from American Type Culture Collection (ATCC; Manassas, VA, USA). The cells were cultured in RPMI 1640 (Thermo Fisher) or DMEM (Thermo Fisher) medium supplemented with 10% fetal bovine serum (FBS, Thermo fisher) and 100 U ml^−1^ penicillin/streptomycin (Thermo Fisher) at 37°C and 5% CO_2_.

### PBMC Isolation and Vγ9Vδ2 T Cell Culture *In Vitro*

PBMCs were isolated from the healthy volunteers’ whole blood (The First Affiliated Hospital of Jinan University), following standard Ficoll−Paque-based (GE Healthcare) density gradient centrifugation protocol. Vγ9Vδ2 T cells were generated with Zoledronate (Sigma-Aldrich) as described (40). Briefly, isolated PBMCs were suspended in RPMI 1640 medium supplemented with 10% FBS, 100 U ml^−1^ recombinant human interleukin-2 (Peprotech), and zoledronate (50 μM). The PBMCs were seeded into 24 well plates, followed by routine culture procedures for 10 days before further tests. Vγ9Vδ2 T cells were maintained at a cell density of 1×10^6^ ml^−1^. When ratio of Vγ9Vδ2 T cells out of total CD3^+^ cells reached 90%, they could be used in experiments. Vγ9Vδ2 T cells were characterized with PerCP-conjugated antihuman TCR Vδ2 (BioLegend) and V500-conjugated antihuman CD3 (BD Biosciences) *via* flow cytometry. In some experiments, the Vγ9Vδ2 T cells were further purified by negative selection with EasySep™ Human Gamma/Delta T Cell Isolation Kit (STEM CELL), and the purity of enriched Vγ9Vδ2 T cells was validated by flow cytometry and was generally higher than 98%.

### Cytotoxic Assay

Breast cancer cells were first labeled with 0.5 μM green fluorescence dye CFSE (Thermo Fisher), followed by incubation with Vγ9Vδ2 T cells according to the designated E (effector, Vγ9Vδ2 T): T (target, cancer cell) ratios (0:1, 5:1, and 15:1) at 37°C in a humidified atmosphere with 5% CO_2_. After 6 hours, cells were harvested and stained with PI (SUNGENE BIOTECH) for 10 minutes at room temperature. The percentage of dead target cells (CFSE^+^ PI^+^) out of the total target cells was identified *via* flow cytometry. In some experiments, breast cancer cells (MCF-7 and MDA-MB-231) and Vγ9Vδ2 T cells were treated with 0.5 mM 1-Methyl-DL-tryptophan (Sigma-Aldrich), 100 nM Linrodostat (Selleck) or with 10 μg ml^−1^ PD-L1 (CD274) antibody (Selleck) for 6 or 12 hours. Co-incubation meant that residual 1-MT or anti-PD-L1 in the medium was not washed out and remained in the subsequent tumor killing assays.

### *In Vitro* Assays

For apoptosis detection, tumor cell lines (MDA-MB-231, MCF-7) were treated with vehicle, 0.5 mM 1-MT in the presence of RPMI 1640 medium supplemented with 10% fetal bovine serum for 6, or 12 hours. Cells were then harvested and stained with PI for 15 minutes at room temperature. The percentage of dead target cells (PI^+^) out of the total number of target cells was determined with flow cytometry.

### Human Patient Samples

Thirty patients who were diagnosed with breast cancer (TNBC) by pathological examination were recruited from outpatient clinics in the First Affiliated Hospital of Jinan University. The patients’ information was shown in **Supplementary Table 1**. Thirty sex- and age-matched healthy volunteers were recruited from the medical staff at the First Affiliated Hospital of Jinan University. PBMCs were isolated from their whole blood, following the standard Ficoll–Paque-based density gradient centrifugation protocol.

### Flow Cytometry

Approximately 10^5^ to 10^6^ human Vγ9Vδ2 T cells were incubated with specific antibodies for 20 minutes at 4°C in the dark for cell surface staining. In some experiments, fresh PBMCs from patients and healthy donors were stimulated with 50 ng/mL phorbol 12-myristate 13-acetate (Sigma-Aldrich) and 1 μg/mL ionomycin (Sigma-Aldrich) in the presence of Golgi Stop (BD Biosciences) for 4 hours, then fixed and permeabilized (BD Biosciences) with antibodies for another 40 minutes at 4°C in the dark, all procedures followed the manufacturer’s recommendations (BD Biosciences). The following antibodies were used: PerCP-conjugated anti-human TCR Vδ2 (BioLegend), V500-conjugated anti-human CD3 (BD Biosciences), PE-conjugated anti-human CD28 (BioLegend), PE/Cy7-conjugated anti-human CD45RA (BioLegend), APC-conjugated anti-human CD27 (eBioscience), Pacific Blue™-conjugated anti-human CD279 (BioLegend), APC-conjugated anti-human Tim-3 (BioLegend), APC-conjugated anti-human TIGIT (BioLegend), FITC-conjugated anti-human IFN-γ (BioLegend), APC-conjugated anti-human TNF-α (BioLegend), Pacific Blue™-conjugated anti-human granzyme B (BioLegend), PE/Cy7-conjugated anti-human perforin (BioLegend), and PE/Cy7-conjugated anti-human CD314 (BioLegend). PerCP-conjugated anti-human CD8a (Biolegend), FITC-conjugated anti-human Vδ1 (Miltenyi Biotec), Brilliant Violet 421™-conjugated anti-human CD274 (Biolegend), APC-conjugated anti-human MICA/MICB (Biolegend), and PerCP-conjugated anti-human CD4 (Biolegend). All flow cytometry data analysis was conducted with FlowJo (FLOWJO, LLC) software.

### Cytokine Secretion Assay

The effector cells (E, Vγ9Vδ2 T cells) were co-cultured with target cells (T, MCF-7, MDA-MB-231) for 6 or 12 hours at an E:T ratio of 1:0, 5:1, 15:1 and the medium supernatant was detected for the levels of cytokine secretion. In some experiments, breast cancer and Vγ9Vδ2 T cells were treated with 0.5 mM 1-Methyl-DL-tryptophan (1-MT) for 6 or 12 hours. The concentration of TNF-α (TB care health), IFN-γ (TB care health), Perforin (Dakewe), and Granzyme B (Dakewe) were detected using enzyme-linked immunosorbent assay.

### NOD/Scid Mice Tumor Models and *In Vivo* Assessment

Non/obese diabetic severe combined immunodeficient (NOD-scid) mice were purchased from Beijing Vital River Laboratory Animal Technology Co., Ltd. All mice were fed with autoclaved food and water. All animal protocols were approved by the Jinan University Institutional Laboratory Animal Care and Use Committee (approval number, IACUC-20190115-04). Mice were used between 5 weeks to 8 weeks of age. 5×10^6^ MCF-7 or 1×10^6^ MDA-MB-231 tumor cells in 200 μL of phosphate buffer solution (PBS) were subcutaneously injected. Engrafted mice were randomized into four groups (n=5 per group): PBS, Vγ9Vδ2 T cell (Vδ2), αPD-L1 (Selleck), and combination of Vγ9Vδ2 T cells and αPD-L1. At the indicated time for each experiment, 10^7^ Vγ9Vδ2 T cells or αPD-L1 antibodies (250 μg per mice) in 200 μL of PBS were adoptively systemically transferred into tumor-bearing (50 mm^3^) mice by tail vein injection. In order to analyze the efficiency of antitumor immunity of Vγ9Vδ2 T cells and αPD-L1, tumor size was recorded until day 39. In the administration of 1-MT in drinking water, 1-MT (Sigma-Aldrich) was prepared at 5 mg ml^−1^ (pH 9–10) in water as previously described (48). Engrafted mice were randomized into four groups: PBS, Vγ9Vδ2 T cell, 1-MT, and combination of Vγ9Vδ2 T cells and 1-MT group. At the indicated time for each experiment, 10^7^ Vγ9Vδ2 T cells in 200 μL of PBS were adoptively systemically transferred into tumor-bearing (50 mm^3^) mice by tail vein injection. Mice (control group) drank 4−5 mL H_2_O/day, similar to the sum of water consumed without drug. 1-MT was administered in the drinking water for 3 weeks, starting at day 7 after tumor challenge (the fresh drinking water containing L-1MT was replaced for four times at 4-day intervals). Tumor size was measured every 2 or 3 days with a caliper, and the tumor volume was calculated using the following equation: $TV=\frac{L\times W^{2}}{2}$(W=width, and L=length). Mice were sacrificed when the tumor volume reached a size of 1500 mm^3^. All animal experiments were independently repeated three times.

### Western Blotting

MDA-MB-231 or MCF-7 cells were treated with the culture medium of Vγ9Vδ2 T cells or IFN-γ for 6, and 12 hours, and then pretreatment tumor cell lines were lysed in RIPA buffer (1 mM PMSF and complete protease inhibitor cocktail, Beyotime; Bimake) at ice for 30 minutes. After centrifugation at 13000 rpm for 15 minutes at 4°C, the supernatants were boiled for 10 minutes at 100°C. Samples were run on 12% SDS-PAGE gel, and then transferred to PVDF membranes, followed by blockade with 5% bovine serum albumin (BSA) for 2 hours at room temperature. PVDF membranes were incubated with indicated primary antibody overnight at 4°C. The following primary antibodies were used:

Primary antibodies: p-STAT1 (CST), p-NF-κB (CST), IDO1 (CST), and β-actin (CST) were purchased from Cell Signaling Technology. After the membranes were washed in TBST for five times, they were incubated with HRP-linked secondary antibody at a dilution of 1:3,000 (CST) at room temperature for 2 hours and were detected with the Bio-Rad ChemiDoc MP Gel imaging system (Bio-Rad).

### Immunohistochemistry Staining

Paraffin-embedded clinical Luminal A and TNBC specimens were obtained from the First Affiliated Hospital, Jinan University (Guangzhou, China). PD-L1 and IDO1 staining was performed according to the protocol described in previous study (41). In brief, Tissues were incubated with anti-PD-L1 (CST) and anti-IDO1 (CST) antibody at 4°C overnight. Sections were rinsed with PBS and incubated with goat anti-rabbit antibody for 1 hour. Finally, slides were further developed with DAB substrate and then counterstained with Mayers hematoxylin.

### Enzyme-Linked Immunosorbent Assay (ELISA)

Cell culture supernatant were collected, and the concentration of kynurenine was measured with ELISA kits in accordance with the manufacturer’s instructions (Bioswamp). In brief, Vγ9Vδ2 T cells (effector) were co-incubated with breast cancer cells (target) at different effector:target (E:T) ratios (0:1, 1:1, 5:1, and 15:1) at 37°C for 12 hours (tumor cells number = 2.5×10^5^). In some experiments, the breast cancer and Vγ9Vδ2 T cells were treated with 0.5 mM 1-methyl-DL-tryptophan (Sigma-Aldrich) for 12 hours. Co-incubation meant that residual 1-MT in the medium was not washed out and remained in the subsequent experiment. Lastly, the cell culture mixture was centrifuged, and the culture supernatant was collected to clean tubes for standard kynurenine measurement.

### Data Availability

The single-cell RNA-sequencing data that support the findings of this study were available from Expression Atlas: https://www.ebi.ac.uk/gxa/sc/experiments. The data of single-cell RNA-sequencing was obtained from Single-cell RNA-seq enables comprehensive tumor and immune cell profiling in primary breast cancer (42), and illustrated by the tool of Single Cell Expression Atlas (43). The levels of *PD-L1* and *IDO1* in breast cancer were displayed as CPM (counts per million) and broken down into four different, logarithmic color ranges: Grey spot: expression level was below cutoff (0.1 CPM) or undetected; Light blue spot: expression level was low (between 0.1 to 10 CPM); Medium blue spot: expression level was medium (between 11 to 1000 CPM); Dark blue spot: expression level was high (more than 1000 CPM). The RNA-Seq dataset that support the conclusions of this article are available from GEPIA: http://gepia2.cancer-pku.cn/#index. The RNA-Seq datasets GEPIA was based on the UCSC Xena project (http://xena.ucsc.edu/), which were computed by a standard pipeline. Linear regression analysis between PD-L1 (CD274) and IDO1 in human breast cancer samples from the TCGA dataset (BRCA cases, *n*=1085), and linear regression analysis were performed using Pearson (44). GEPIA was also applied to evaluate the overall survival (OS) and diseases-free survival (DFS) of patients with high or low CD274 (PD-L1) expression in Luminal A [*n* (high)=205, *n* (low)=205] and TNBC [*n* (high)=67, *n* (low)=67]. The cutoff was defined as: Group Cutoff (Median), Cutoff-High (%) and Cutoff-Low (%) =50, and survival analysis based on the expression status of PD-L1 signature and plot a Kaplan-Meier curve. Box plots showed the level of signatures gene set in cancer tissues and para-cancerous tissue with TNBC and Luminal A subtypes. Signatures Gene Set: Naïve T-cell (*CCR7, LEF1, TCF7*, and *SELL*), effector T-cell (*CX3CR1, FGFBP2*, and *FCGR3A*), central memory T-cell (*CCR7, SELL*, and *IL7R*), exhausted T-cell (*HAVCR2, TIGIT, LAG3*, and *PDCD1*), and resting Treg T-cell (*FOXP3* and *IL2RA*), or the specific gene indicated as IDO1. (TNBC, *n*=135; luminal A, *n*=415; para-cancerous tissues, *n*=291). The Log2^FC^ Cutoff = 1, and the p-value Cut off = 0.01. For pathological stage plot analysis, GEPIA used major stage (yes) or sub-stage (no) for plotting, Log scale defined as log_2_(TPM+1) for log-scale by GEPIA database.

### Statistical Analysis

Statistical analyses were performed using GraphPad Prism 6.0 (GraphPad Software). Where indicated, data were analyzed for statistical significance and reported as p-values. Data were analyzed by two-tailed Student’s t test when comparing means of two independent groups and two-way ANOVA when comparing more than two groups. For sample sizes of *n* > 3, the data distribution was first checked using a Kolmogorov–Smirnov test. If the data fitted a normal distribution, a two-tailed unpaired Student’s *t*-test was used when variances were similar, whereas a two-tailed unpaired Student’s *t*-test with Welch’s correction was used when variances were different. If the data did not fit a normal distribution, a Mann–Whitney U-test was used. *P* < 0.05 was considered statistically significant (*, *P* < 0.05; **, *P* < 0.01; ***, *P* < 0.001; and ****, *P* < 0.0001, *n.s.*, not significant). Data were presented as the mean ± SD.

## Results

### IDO1 and PD-L1 Were Co-Expressed in Triple-Negative Breast Cancer Samples

**Figure 1:**
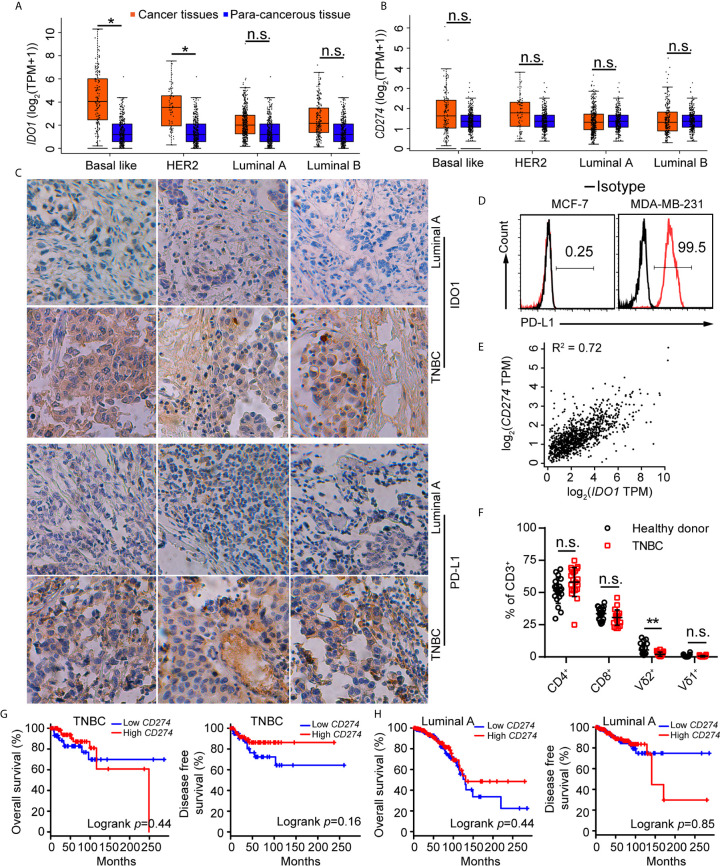
IDO1 and PD-L1 were co-expressed in triple-negative breast cancers. **(A, B)** Expression of *IDO1* and *PD-L1* in breast cancers from the GEPIA dataset (Basal like, Cancer tissues (*n*=135); HER2, Cancer tissues (*n*=66); Luminal A, Cancer tissues (*n*=415); Luminal B, Cancer tissues (*n*=194); para-cancerous tissues, *n*=291). Cancer tissues (orange box) labeled as “tumor” were compared with para-cancerous tissues (blue box) labeled as “normal”. Immunohistochemistry **(C)** for IDO1 and PD-L1 expression in cancer tissues with luminal A and TNBC (*n*=3). **(D)** Representative histograms of PD-L1 expression by MCF-7 (luminal A) and MDA-MB-231 (TNBC) cells. **(E)** Correlation between *PD-L1* and *IDO1* expression in human breast cancer samples from the GEPIA (*n*=1085; R^2^ = values by linear regression). **(F)** Dot plots showing T cell subsets isolated from healthy or patient donors with TNBC PBMCs, expressed as percentage of CD3^+^ cells (*n*=20). **(G)** Overall survival and disease-free survival of triple-negative breast cancer patients with high or low *CD274* (*PD-L1*) expression (*n* (high)=67, *n* (low)=67) as defined by the median. Compared by log-rank (Mantel-Cox) test (GEPIA data base). **(H)** Overall survival and disease-free survival of luminal A breast cancer patients with high or low *CD274* (*PD-L1*) expression (*n* (high)=205, *n* (low)=205) as defined by the median. Compared by log-rank (Mantel−Cox) test (GEPIA data base). Unpaired Student’s *t*-test (F). Significance was set to *P* < 0.05 and represented as *P < 0.05, **P < 0.01, ***P < 0.001, and ****P < 0.0001, *n.s.*, not significant.

Tumor cells evaded immune surveillance by expressing immunosuppressive factors, such as PD-L1 and IDO1 (15). We analyzed the levels of *PD-L1* and *IDO1* in all subtypes of breast cancer and found that expression of *IDO1* in cancer tissues were higher in para-cancerous tissues at basal like (TNBC) positive breast cancer (**Figure 1A**) from the Gene Expression Profiling Interactive Analysis (GEPIA) (44). Compared with other subtypes, Luminal A cancer had the lowest expression of *PD-L1* (**Figure 1B**) and *IDO1*, suggesting that IDO1 may play important role in TNBC but not Luminal A cancers. We further examined the expression of *IDO1* and *PD-L1* in breast cancer patients by analyzing single-cell RNA-sequencing data from The Single Cell Expression Atlas. The results showed that the level of *PD-L1* and *IDO1* were higher in TNBC than that in Luminal A positive breast cancer (42, 43) (**Supplementary Figure 1**). The results of immunohistochemical staining further validated that protein levels of PD-L1 and IDO1 were higher in patients with TNBC than that with Luminal A subset (**Figure 1C**). The most commonly used representative TNBC cell line was MDA-MB-231, and the most commonly used Luminal A cell line was MCF7. Flow cytometric analysis confirmed that PD-L1 was highly expressed on MDA-MB-231 cell line (**Figure 1D**). The RNA-sequencing data from the Gene Expression Profiling Interactive Analysis (GEPIA) (44) revealed that expression of *PD-L1* was positively correlated with that of *IDO1* in breast cancer patients (**Figure 1E**). Moreover, the percentage of Vγ9Vδ2^+^CD3^+^ T cells in PBMCs of TNBC patients was significantly lower than that of healthy controls (**Figure 1F**). However, the level of PD-L1 has no significant correlation with overall survival advantage and diseases-free survival for patients with TNBC and Luminal A subtypes (**Figures 1G, H**). These data indicated that IDO1 and PD-L1 expressed and were significantly higher in TNBC than in Luminal A positive breast cancer. However, the biomarkers to predict responses to ICIs in breast cancer patients remain debatable and urgently need to be clarified.

### Immune Checkpoint Receptors Were Highly Expressed on Vγ9Vδ2 T Cells in Triple-Negative Breast Cancer Patients

**Figure 2:**
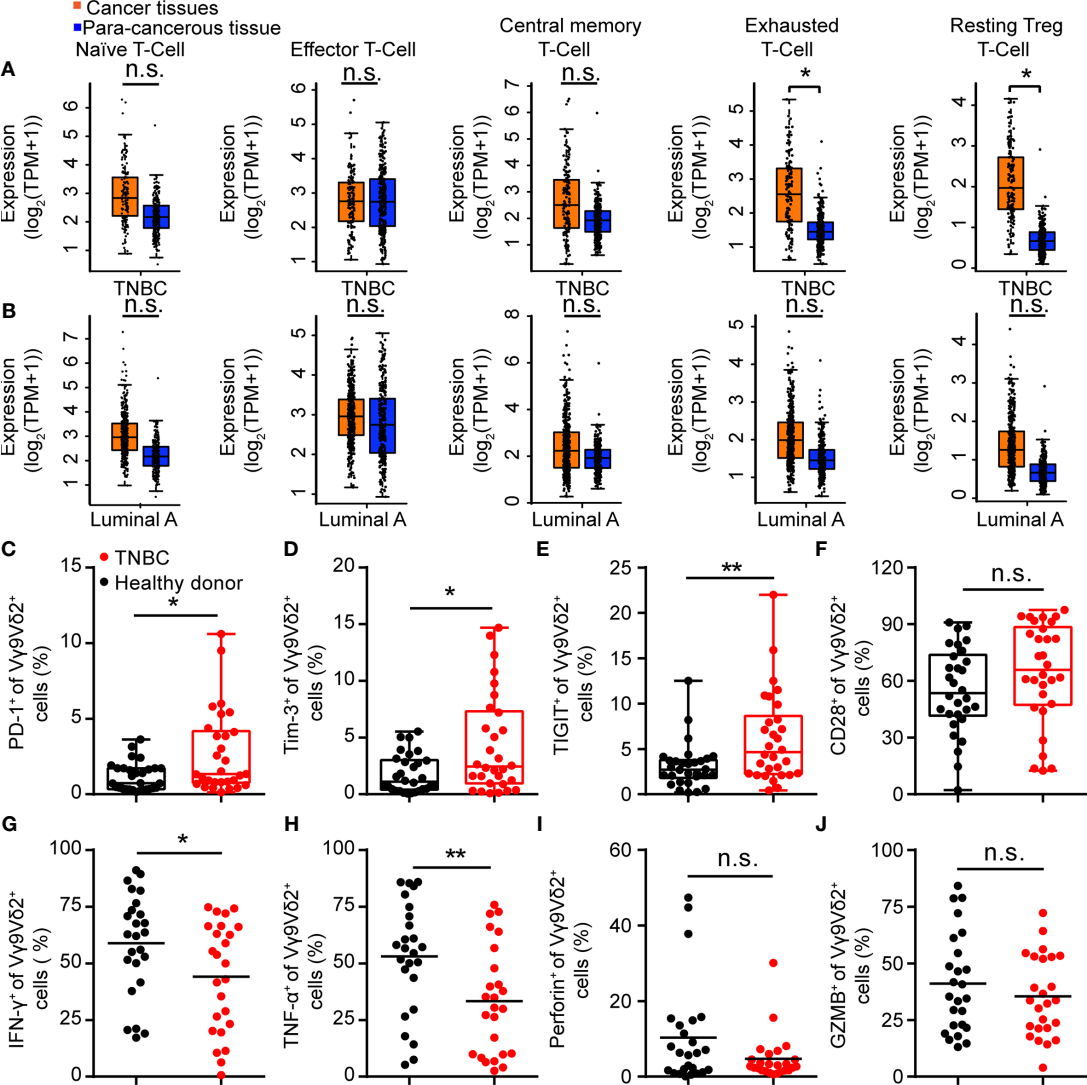
Immune checkpoint receptors were highly expressed on Vγ9Vδ2 T cells in triple negative breast cancer patients. **(A, B)** Expression of T cell function-associated genes in TNBC and luminal A cancers from the GEPIA dataset (TNBC, *n*=135, luminal A, *n*=415). Cancer tissues (orange box) were compared with para-cancerous tissues (blue box, *n*=291). Signatures Gene Set: Naïve T-cell (*CCR7, LEF1, TCF7*, and *SELL*), effector T-cell (*CX3CR1, FGFBP2*, and *FCGR3A*), central memory T-cell (*CCR7, SELL*, and *IL7R*), exhausted T-cell (*HAVCR2, TIGIT, LAG3*, and *PDCD1*), and resting Treg T-cell (*FOXP3* and *IL2RA*). The method for differential analysis was one-way ANOVA, using disease state (Tumor or Normal) as variable for calculating differential expression **(A, B)**. **(C–F)** Expression of PD-1^+^ **(C)**, Tim-3^+^ **(D)**, TIGIT^+^ **(E)**, and CD28^+^ **(F)** on Vγ9Vδ2 T cells in healthy donors (*n*=30) and triple negative breast cancer patients (*n*=30). **(G–J)** Percentage of Vγ9Vδ2 T cells producing IFN-γ^+^ **(G)**, TNF-α^+^ **(H)**, Perforin^+^ **(I)**, and Granzyme B^+^ **(J)** in total Vγ9Vδ2 T cells were from patients with TNBC (*n*=25) and healthy donors (*n*=25), and stimulated with 50 ng/mL phorbol 12-myristate 13-acetate (PMA) and 1 μg/mL ionomycin (Ion) in *vitro*. Unpaired Student’s *t*-test **(F–H, J)**; Mann−Whitney test **(C–E, I)**. Significance was set to *P* < 0.05 and represented as *P < 0.05, **P < 0.01, ***P < 0.001, and ****P < 0.0001, *n.s.*, not significant.

Vγ9Vδ2 T cells are another type of cytotoxic T cells important for tumor surveillance in human, besides CD8^+^ T lymphocytes. When exploring the immune status of breast cancer patients, we found that immune exhaustion and T reg phenotypes in TNBC samples were higher than that in luminal A subtypes from the GEPIA data (44) (**Figures 2A, B**). In order to investigate the status of Vγ9Vδ2 T cells in human TNBC, we recruited 30 patients with advanced TNBC and 30 healthy volunteers. The expression of immune checkpoint receptors on Vγ9Vδ2 T cells from the peripheral blood mononuclear cells (PBMCs) was analyzed. The characteristics of the healthy volunteers and patients were summarized in **Table 1**. The TNBC Vγ9Vδ2^+^ T cells showed immune exhaustion phenotype as represented by the increased expression of PD-1, TIGIT, and Tim-3, while cell surface CD28 expression was insignificantly changed (**Figures 2C–F** and **Supplementary Figure 2**). The Vγ9Vδ2 T cells from the TNBC patients produced less amounts of IFN-γ and TNF-α, whereas Granzyme B and Perforin expression were marginally reduced, when stimulated with phorbol 12-myristate 13-acetate and ionomycin *in vitro*, compared with those from healthy donors (**Figures 2G–J** and **Supplementary Figure 3**). These results revealed that the quality of Vγ9Vδ2 T cells in TNBC patients was attenuated. This phenomenon might be due to the increased ICI level on these cells.

**Table 1:** Information of TNBC patients and healthy donors.

Patient characteristics					Healthy donor characteristics		
Case ID	Gender	Age	Types of cancer	Stage at diagnosis	Case ID	Gender	Age
1	female	61	Triple-negative breast cancer	IV	1	female	51
2	female	48	Triple-negative breast cancer	IV	2	female	40
3	female	63	Triple-negative breast cancer	IV	3	female	66
4	female	58	Triple-negative breast cancer	IV	4	female	65
5	female	46	Triple-negative breast cancer	IV	5	female	55
6	female	64	Triple-negative breast cancer	IV	6	female	52
7	female	67	Triple-negative breast cancer	IV	7	female	48
8	female	54	Triple-negative breast cancer	III	8	female	68
9	female	59	Triple-negative breast cancer	III	9	female	57
10	female	38	Triple-negative breast cancer	IV	10	female	68
11	female	44	Triple-negative breast cancer	IV	11	female	32
12	female	57	Triple-negative breast cancer	IV	12	female	77
13	female	55	Triple-negative breast cancer	IV	13	female	56
14	female	63	Triple-negative breast cancer	III	14	female	55
15	female	65	Triple-negative breast cancer	IV	15	female	63
16	female	67	Triple-negative breast cancer	IV	16	female	67
17	female	70	Triple-negative breast cancer	IV	17	female	69
18	female	66	Triple-negative breast cancer	IV	18	female	55
19	female	54	Triple-negative breast cancer	IV	19	female	51
20	female	68	Triple-negative breast cancer	IV	20	female	49
21	female	55	Triple-negative breast cancer	III	21	female	55
22	female	47	Triple-negative breast cancer	III	22	female	75
23	female	33	Triple-negative breast cancer	III	23	female	43
24	female	67	Triple-negative breast cancer	IV	24	female	37
25	female	82	Triple-negative breast cancer	IV	25	female	39
26	female	65	Triple-negative breast cancer	IV	26	female	78
27	female	54	Triple-negative breast cancer	IV	27	female	65
28	female	39	Triple-negative breast cancer	IV	28	female	63
29	female	47	Triple-negative breast cancer	IV	29	female	59
30	female	66	Triple-negative breast cancer	III	30	female	68

### Vγ9Vδ2 T Cells Could Kill Breast Cancer Cells and Restrain Cancer Growth, But Without Enhanced Function With Anti-PD-L1 Addition

**Figure 3:**
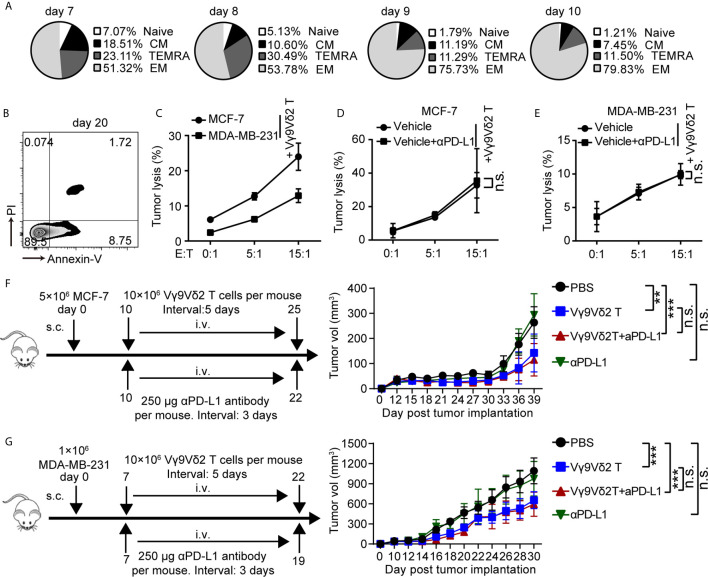
Vγ9Vδ2 T cells could kill breast cancer cells and restrain cancer growth, but without enhanced function with anti-PD-L1 addition. **(A)** Subsets of Vγ9Vδ2 T cells cultured on day 7, 8, 9, and 10. According to the CD45RA and CD27 expression, the Vγ9Vδ2 T lymphocytes were subdivided into: CM (CD27^+^CD45RA^−^), naïve (CD27^+^CD45RA^+^), TEMRA (CD27^−^CD45RA^+^), and EM (CD27^−^CD45RA^−^). **(B)** Survival of Vγ9Vδ2 T cells cultured to day 20. Cell apoptosis (PI^+^ Annexin-V^+^) were analyzed by flow cytometry. **(C)** Cytotoxicity of Vγ9Vδ2 T cells toward breast cancer cells (MCF-7 or MDA-MB-231) at the indicated effector to target ratio (E:T). The percentages of dead cells out of whole target cells were identified as PI^+^ (*n*=5). **(D, E)** Cytotoxicity of Vγ9Vδ2 T cells had no obvious difference at the indicated E:T ratio with MCF-7 or MDA-MB-231 cells (target cells) pretreated with vehicle or anti-PD-L1 (10 μg/mL) for 6 hours (*n*=5). Dead target cells out of the total target cells were recorded. **(F, G)** Schematic protocols of tumor growth model (left). i.v., intravenous; s.c., subcutaneous. Progression of MCF-7 tumors and MDA-MB-231 tumors in mice was assessed in NOD/scid mice. *n*=5 mice per group. Experiments were independently repeated three times **(F, G)**. Vγ9Vδ2 T cells from healthy donors were expanded with ZOL. Data represent mean ± SD **(C–G)**. Unpaired Student’s *t*-test **(C–E)**; two-way analysis of variance (ANOVA) **(F, G)**. Significance was set to *P* < 0.05 and represented as *P < 0.05, **P < 0.01, ***P < 0.001, and ****P < 0.0001, *n.s.*, not significant.

Recent clinical studies demonstrated the safety and efficacy of allogeneic Vγ9Vδ2 T-cell immunotherapy (32). On this basis, we hypothesized that combination of Vγ9Vδ2 T cells and anti-PD-L1 might be important for TNBC immunosurveillance and might help restrain cancer growth. To address this question, we expanded Vγ9Vδ2 T lymphocytes by culturing human PBMCs from healthy donors *in vitro*. Zoledronate (ZOL)-expanded Vγ9Vδ2 T cells typically accounted for more than 90% of the cultured PBMCs (**Supplementary Figure 4A**). These cells were mainly effector memory T cells (CD27^−^CD45RA^−^), which showed efficient cytotoxic activity and the potential for cytokine production (**Figure 3A** and **Supplementary Figure 4B**). The purity of the enriched Vγ9Vδ2 T cells was validated by flow cytometry and was generally higher than 95% (**Supplementary Figure 4C**). Flow cytometric analysis confirmed that the ZOL-expanded Vγ9Vδ2 T lymphocytes *in vitro* induced NKG2D expression, whereas the level of PD-1 marginally increased, suggesting that ZOL-expanded Vγ9Vδ2 T cells were suitable for further adoptive immunotherapy (**Supplementary Figures 4D, E**). The frequency of viable Vγ9Vδ2 T cells (annexin-V^−^/PI^−^), early apoptotic cells (annexin-V^+^/PI^−^), and late apoptotic cells (annexin-V^+^/PI^+^) was analyzed by flow cytometry (**Figure 3B**). The ZOL-expanded Vγ9Vδ2 T cells showed few annexin-V^+^/PI^+^ population when cultured to day 20, thereby suggesting that long-term-expanded Vγ9Vδ2 T cells had sustained anti-apoptotic ability *in vitro*. We performed killing assay by using breast cancer cells co-cultured with Vγ9Vδ2 T cells *in vitro* to investigate whether Vγ9Vδ2 T cells could inhibit the growth of breast cancer cells. The proportion of apoptotic cells in MCF-7 (Luminal A) was generally higher than that in MDA-MB-231 (TNBC) when they were treated with Vγ9Vδ2 T cell alone; meanwhile, the combination of Vγ9Vδ2 T cells and anti-PD-L1 could not further enhance the killing efficacy (**Figures 3C–E** and **Supplementary Figures 5A, B**). In a tumor-bearing mouse model, no obvious difference of tumor size shrinkage was observed under the therapy of Vγ9Vδ2 T cells combined with anti-PD-L1, compared with that of Vγ9Vδ2 T cells alone (**Figures 3F, G**). In summary, these results showed that Vγ9Vδ2 T cells could kill breast cancer cells; however, anti-PD-L1 antibody did not obviously contribute to promote the antitumor function of these cells.

### IDO1 Inhibitor 1-MT Enhanced the Antitumor Efficacy of Vγ9Vδ2 T Cells

**Figure 4:**
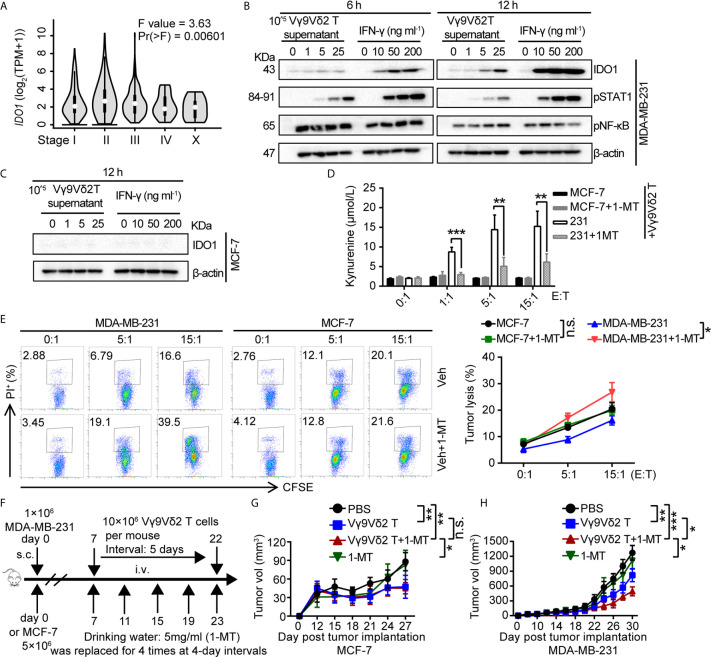
1-MT enhanced the antitumor efficacy of Vγ9Vδ2 T cells. **(A)** Expression of *IDO1* in BRCA at different pathological stages, (BRCA, *n*=1085). **(B)** MDA-MB-231 cells were treated with the culture medium of Vγ9Vδ2 T cells or IFN-γ for 6, and 12 hours. Western blot to detect IDO1, p-STAT1, and p-NF-κB expression; β-actin was used as the loading control. **(C)** MCF-7 cells were treated with culture supernatant of Vγ9Vδ2 T cells or IFN-γ for 12 hr. Western blot to detect IDO1 level; β-actin was used as the loading control. **(D)** Kynurenine was the metabolite of L-tryptophan catalyzed by IDO1 enzyme, and its level in the culture supernatant of MCF-7 and MDA-MB-231 cells cocultured with Vγ9Vδ2 T cells treated with or without 1-MT was shown. The amounts of kynurenine were measured by ELISA kits. **(E)** IDO1 inhibitor 1-MT facilitated the cytotoxicity of Vγ9Vδ2 T cells toward MDA-MB-231 cells, but not MCF-7 cells. MCF-7 or MDA-MB-231 cells (target) were co-cultured with Vγ9Vδ2 T cells (effector) with 1-MT or vehicle for 6 hours. The percentage of dead cells out of total target cells was shown. **(F–H)** Schematic protocols of tumor growth model (left). *n*=5 mice per group. Experiments were independently repeated three times **(H, I)**. Results in **(C, D)** were representative blots from 2 to 3 independent experiments. Vγ9Vδ2 T cells from healthy donors were expanded with ZOL. Data represent mean ± SD **(E, F, H, I)**. Unpaired two-tailed Student’s *t*-test **(E, F)**; one-way ANOVA using the disease state (tumor or normal) as variable for calculating the differential expression of IDO1 **(A)**; The method for *IDO1* expression analysis was one-way ANOVA by using the pathological stage as variable for calculating differential expression **(B)**; two-way ANOVA **(H, I)**. Significance was set to *P* < 0.05 and represented as *P < 0.05, **P < 0.01, ***P < 0.001, and ****P < 0.0001, *n.s.*, not significant.

Based on these observations, we assumed that IDO1 co-expressed with PD-L1 might be involved in regulating the antitumor activity of Vγ9Vδ2 T cells. Accordingly, we examined the level of *IDO1* in breast cancer samples by using RNA-sequencing data from GEPIA (44). The analysis results showed that it differently expressed in different pathological stages (**Figure 4A**). We detected the expression of pSTAT1 and found that pSTAT1 expression positively correlated with IDO1 expression and was IFN-γ dose-dependent in MDA-MB-231 cell lines (**Figure 4B**). The expression of IDO1 enzyme in MDA-MB-231 cells was induced by IFN-γ and the culture supernatant of activated Vγ9Vδ2 T cells, while it was barely detectable in MCF-7 cells (**Figure 4C**). This finding suggested that the upregulated expression of IDO1 in MDA-MB-231 cells might depend on the IFN-γ-pSTAT1 signaling pathway (2). Then we conducted a killing experiment *in vitro* to further validate whether IDO1 enzyme activity was involved in regulating the antitumor function of Vγ9Vδ2 T cells. The MDA-MB-231 cells were co-cultured with Vγ9Vδ2 T cells according to the designated E (effector, Vγ9Vδ2 T cell): T (target, tumor cell) ratios. The result showed that kynurenine level were significantly reduced by IDO1 enzyme inhibitor 1-MT (**Figure 4D**). The proportion of apoptotic MDA-MB-231 cells was higher in the group treated with Vγ9Vδ2 T cells and 1-MT or Lindrost (BMS-986205) than that in the group of Vγ9Vδ2 T cell treatment alone (**Figure 4E**; **Supplementary Figure 6A**). Moreover, 1-MT or Lindrostat treatment alone (without Vγ9Vδ2 T cells) did not increase the cell death (**Supplementary Figures 6B, C**). The NOD-scid immuno-deficient mice were inoculated with MDA-MB-231 cells to evaluate the therapeutic effects of Vγ9Vδ2 T cells and 1-MT for breast cancer. Seven days later, the mice were treated with Vγ9Vδ2 T cells and/or 1-MT. The results showed that the volume of tumors in the group treated with Vγ9Vδ2 T cells combined with 1-MT was significantly smaller than that of PBS group and groups treated with Vγ9Vδ2 T cell alone or 1-MT alone (**Figures 4F–H**). These data indicated that human Vγ9Vδ2 T cells could kill MDA-MB-231 cells and restrain tumor growth, and the IDO enzyme inhibitor themselves could not kill breast cancer cells efficiently, but could enhance the antitumor efficacy of Vγ9Vδ2 T cells.

### Treatment With 1-MT Promoted Perforin Production of Vγ9Vδ2 T Cells Under MDA-MB-231 Cell Stress

**Figure 5:**
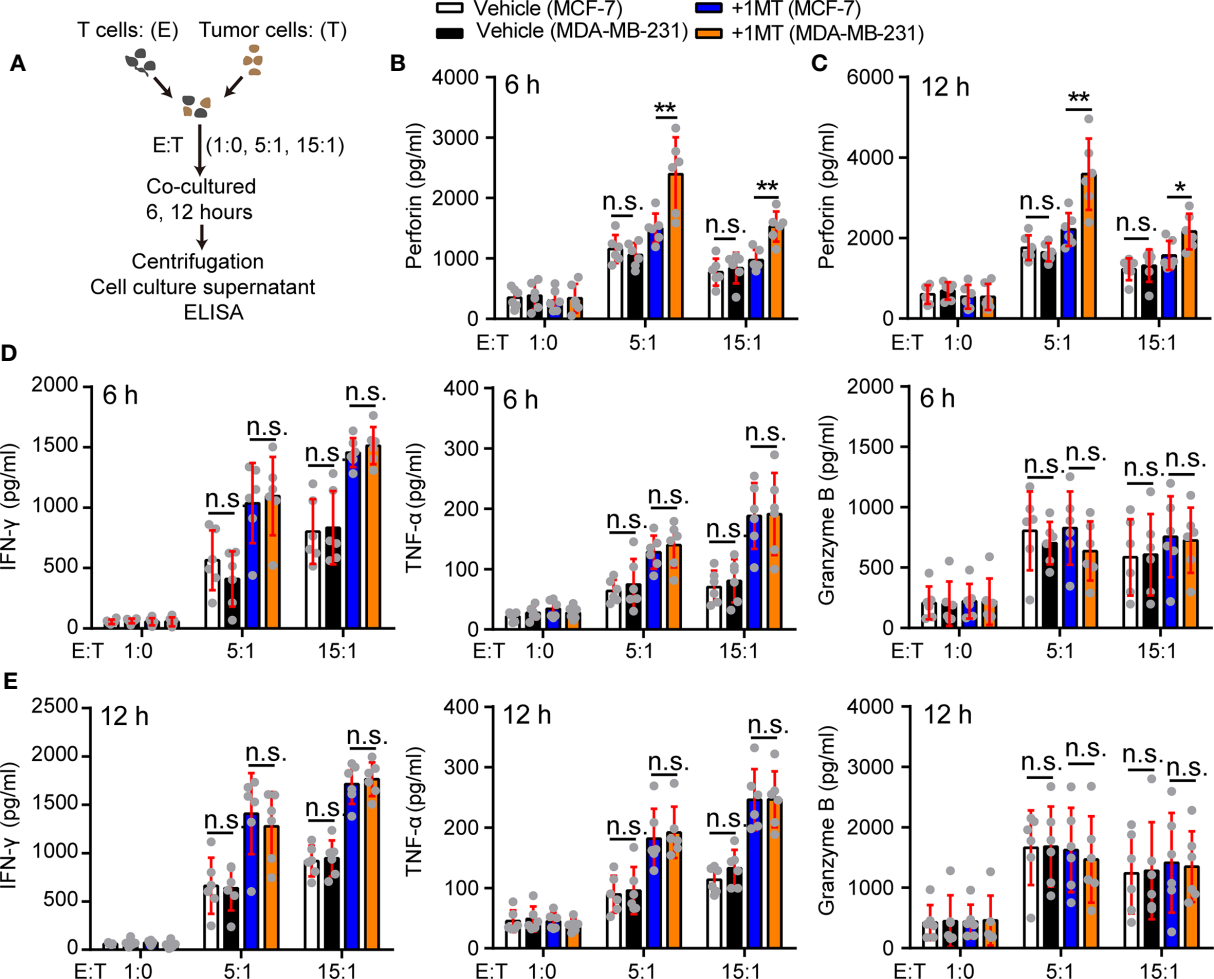
Treatment with 1-MT and MDA-MB-231 cells enhanced perforin production of Vγ9Vδ2 T cells. **(A)** Experimental approach. **(B–E)** The levels of cytokines, released by Vγ9Vδ2 T cells, were measured by ELISA after Vγ9Vδ2 T cells from healthy donors were incubated with MCF-7 or MDA-MB-231 cells for 6 or 12 hours in the presence or absence of 1-MT (500 μM) (*n*=6). Data represent mean ± SD; unpaired Student’s *t*-test **(B–E)**. Significance was set to *P* < 0.05 and represented as *P < 0.05, **P < 0.01, ***P < 0.001, and ****P < 0.0001, *n.s.*, not significant.

How did 1-MT enhance the antitumor function of Vγ9Vδ2 T cells in our experiment system? To address this question, we took advantage of killing system: the MDA-MB-231 or MCF-7 cells were co-cultured with Vγ9Vδ2 T cells with or without 1-MT, and the cytokine secretion in the supernatant of Vγ9Vδ2 T cells was analyzed (**Figure 5A**). The perforin production of Vγ9Vδ2 T cells treated with MCF-7 and 1-MT was not significantly more than that of MCF-7 treatment alone. However, the production of perforin was more significantly induced in the supernatant of Vγ9Vδ2^+^ T cells treated with MDA-MB-231 cells combined with 1-MT, compared with that of MDA-MB-231 treatment alone (**Figures 5B, C**). We found that Vγ9Vδ2^+^ T cells in above co-culture system could produce cytotoxic cytokines IFN-γ, TNF-α and Granzyme B; however, no significant enhancement was observed with 1-MT treatment combination (**Figures 5D, E**). These results showed that blocking the IDO1 enzyme enhanced the cytotoxicity of Vγ9Vδ2 T cells through promoting perforin production. γδ T cells express the activating receptor NKG2D, which endows them with a TCR-independent second activation pathway *via* recognition of NKG2D ligands on tumor cells (45). So, we also detected the NKG2D expression on Vγ9Vδ2 T cells in the same experimental system, but it did not significantly change (**Supplementary Figure 7**). Previous study showed a strong correlation between anticancer activity of Vγ9Vδ2 T cells and MICA/B expression on tumor cells *in vitro* (46, 47). So, we further analyzed the level of MICA/B on MDA-MB-231 and MCF-7 cells, and found that NKG2D’s ligand MICA/B highly expressed on surface of MDA-MB-231 cells, however was barely detectable on MCF-7 cells (**Supplementary Figure 8**). These results indicated that MICA/B might also be involved in the killing of MDA-MA-231 cells by Vγ9Vδ2 T cells.

## Discussion

Approximately 2.26 million newly diagnosed female breast cancer cases were recorded worldwide in 2020 (48). Triple-negative breast cancer is a subtype of breast cancer with the poorest prognosis due to lack of targeted therapies. Immunotherapeutic strategies for treating breast cancers had aroused great interest. Several immunotherapeutic agents had received approval of US Food and Drug Administration (FDA), including vaccines, adoptive cell therapies, oncolytic viruses, and most notably, immune checkpoint blockade (3). Our findings showed that the expression of *PD-L1* was positively correlated with *IDO1* in TNBC patients from the data of GEPIA RNA-sequencing, thereby implying that immunosuppressive effects dominated in this type of tumors and prevented normal T cell function. Gamma delta (γδ) lymphocyte cells infiltrated into most human tumors (49). We detected Vγ9Vδ2 T cells in blood and found that their function was suppressed, and the cell number decreased in TNBC patients. *In vitro* and *in vivo* works showed that human Vγ9Vδ2 T cells could inhibit growth of MDA-MB-231 cells, a type of TNBC cancer cells; however, the combination with anti-PD-L1 treatment could not increase their antitumor capacity. Interestingly Vγ9Vδ2 T cells combined with 1-MT, IDO1 inhibitor, enhanced their antitumor ability. Finally, we found that blocking the IDO1 enzyme enhanced the cytotoxicity of Vγ9Vδ2 T cells by perforin secretion under tumor burden stress.

IDO expression had been illustrated in tumor cells and antigen presenting cells (APC) (human monocyte-derived macrophages and dendritic cell) in a range of human cancer patients and murine cancer models (50, 51). In TNBC subtypes, IDO1 and PD-L1 were upregulated by interferon-γ-secreting T cells in the tumor microenvironment (52). However, previous clinical study showed that there was no clear evidence for benefit of adding navoximod (IDO inhibitor) to atezolizumab (PD-L1) (25). Recent work also indicated that the IDO inhibitor promoted Vγ9Vδ2 T cell’s antitumor response against Ductal Pancreatic Adenocarcinoma Cells (53). The results of those studies suggested that IDO inhibitor probably targeted on human cytotoxic T cells; thus, only T cells infiltrated into tumors would have responded to the IDO inhibitor therapy (54). This finding might explain why the combination of IDO inhibitor and anti-PD-1/PD-L1 therapy did not demonstrate improved survival in many types of cancer patients. In this study, we found that only treatment of Vγ9Vδ2 T cell and 1-MT combination had more significant therapeutic effect and resulted in slower tumor growth in mouse model. This finding suggested that Vγ9Vδ2 T lymphocytes/1-MT combination might have potential applications in therapy of TNBC patients, which would inspire further clinical investigations and eventually benefit cancer patients.

IDO expression was correlated with increased tumor-infiltrating T regulatory cells (T reg); studies had suggested that tumor cells or IDO-expressing APCs could mediate T reg cells to strongly dampen the antitumor responses of T cells (16, 39). The intratumor ratio of infiltrating lymphocytes (TILs) to T reg cells was considered as a marker of favorable immunological responses, which was a possible key factor to control tumor progression (5, 55). Previous work showed that intratumoral γδ T cell numbers were positively correlated with advanced tumor stages, HER2 expression status, FOXP3^+^ cells, and high lymph node metastasis, but inversely correlated with relapse-free survival and overall survival of breast cancer patients (6). However, Andrew J Gentles et al. demonstrated that intra-tumoral γδ T cells emerged as a significant favorable marker in breast cancer survival (10). The possible reason for this contradictory phenomenon was that γδ T cells played different roles in different breast cancer subtypes. T cell exhaustion was a poor responsive status with an upregulated level of ICIs, decreased production of cytotoxic cytokines, and suppressed antitumor efficacy (56). Our data showed that Vγ9Vδ2 T cells in triple-negative breast cancer patients had exhausted phenotype with increased PD-1, TIGIT, and Tim-3 expression and reduced cytokine production. These findings suggested that dysfunctional Vγ9Vδ2 T cells in triple-negative breast cancer patients might be another important factor to accelerate tumor progression besides CD8^+^ T cells.

Cytotoxic T cell exerted antitumor effects *via* cytokine production, such as IFN-γ, TNF-α, granzyme B, and perforin (49), under the specific tumor antigen stimulation. *IDO1* is a classic IFN-γ-inducible gene (57). However, in the analysis of several breast cancer datasets, previous reports showed differences of subtype-specific mRNA and promoter methylation in IDO1, with TNBC/basal subtypes exhibiting low methylation/higher expression phenotype and ER^+^/luminal subtypes demonstrating high methylation/lower expression phenotype (15). Those results suggested that the IDO1 gene in TNBC cells was intrinsically open for transcription. The high expression of IDO1 in TNBC patients could intensively inhibit Vγ9Vδ2 T cells. This phenomenon may explain our results: Vγ9Vδ2 T cells in TNBC patients expressed high levels of ICIs, such as PD-1, Tim3, and TIGIT, and produced less cytokines, such as IFN-γ and TNF-α. The immunosuppressed Vγ9Vδ2 T cells were relieved to produce many cytokines when treated with the IDO inhibitor and restrained tumor growth. We also confirmed that Vγ9Vδ2 T cells treated with the IDO inhibitor promoted perforin production, but not IFN-γ and TNF-α under the tumor stress. However, IDO inhibitor mediated the transcription of perforin needs to be further investigated.

In summary, our data showed that IDO inhibitor facilitated Vγ9Vδ2 T cell antitumor activity. The combination of Vγ9Vδ2 T cells and IDO inhibitor could result in additive and strong effects for suppressing MDA-MB-231 cell growth. Vγ9Vδ2 T cells combined with IDO inhibitor might be a safe and economical approach to treat triple negative breast cancers. The efficacy of Vγ9Vδ2 T cells and IDO blockade therapy in TNBC patients need to be further validated in clinical trials.

## Data Availability Statement

The datasets presented in this study can be found in online repositories. The names of the repository/repositories and accession number(s) can be found below: The Expression Atlas database https://www.ebi.ac.uk/gxa/sc/experiments under the accession number SRP066982.

## Ethics Statement

The studies involving human participants were reviewed and approved by The Institutional Review Board of Jinan University, Guangzhou, P. R. China. The patients/participants provided their written informed consent to participate in this study. The animal study was reviewed and approved by The Jinan University Institutional Laboratory Animal Care and Use Committee.

## Author Contributions

YG and PeL conceived the project and designed the study. PeL, RW, and KL wrote the manuscript, made the figures. PeL and RW performed research. JZ, WY, XZ, YZ, PiL, CZ, and XW provided technical assistance. PeL and YG discussed and interpreted the data.PeL, KL, and RW contributed to manuscript preparation. All authors contributed to the article and approved the submitted version.

## Funding

This work was supported by the National Natural Science Foundation of China (grant 31770964 to YG) and the 111 Project (grant B16021 to YG).

## Conflict of Interest

The authors declare that the research was conducted in the absence of any commercial or financial relationships that could be construed as a potential conflict of interest.

## Supplementary Material

Supplementary Figure 1 The levels of *PD-L1* and *IDO1* in TNBC and Luminal A breast cancers. **(A)** t-SNE plot of all 515 classified cells, demonstrating separation by cell type. Individual cells were colored green for luminal A, yellow for luminal B, blue for HER2, and red for TNBC tumors (The Single Cell Expression Atlas data base). **(B)** Expression levels of *IDO1* and *PD-L1* across 515 single cells illustrated in t-SNE plots. Individual cells were colored red for TNBC, blue for luminal A (The Single Cell Expression Atlas data base).

Supplementary Figure 2 Identification of exhaustion and costimulatory markers in triple negative breast cancer patients. PD-1^+^, Tim-3^+^, TIGIT^+^, and CD28^+^ levels on Vγ9Vδ2^+^ T cells in PBMCs of healthy donors and triple negative breast cancer patients (TNBC) were shown.

Supplementary Figure 3 Cytokine production of Vγ9Vδ2^+^ T cells. Frequency of TNF-α^+^, IFN-γ^+^, Perforin^+^, and Granzyme B^+^ Vγ9Vδ2^+^ T cells in healthy donor and triple negative breast cancer samples were shown.

Supplementary Figure 4 Percentage of NKG2D^+^, PD-1^+^ Vγ9Vδ2 T cells out of the total Vγ9Vδ2 T lymphocyte population. **(A, B)** Representative flow cytometry plots showing the gating strategy to identify lymphocytes including subsets of Vγ9Vδ2 T cells expanded from ZOL. **(C)** Vγ9Vδ2 T cells were further purified by negative selection with EasySep™ Human Gamma/Delta T Cell Isolation Kit. **(D, E)** Vγ9Vδ2 T cells were expanded in *vitro* from the human peripheral blood cells with ZOL. Frequency of NKG2D and PD-1 expression on Vγ9Vδ2^+^ T cells at day 12 was shown.

Supplementary Figure 5 Anti-PD-L1 antibody could not further enhance the antitumor efficacy of Vγ9Vδ2 T cells. **(A)** Cytotoxicity of Vγ9Vδ2 T cells toward MCF-7 or MDA-MB-231 cell lines at the indicated ratio of effector to target (E:T). Frequency of dead cells out of whole target cells were showed as PI^+^. **(B)** Cytotoxicity of Vγ9Vδ2 T cells had no obvious difference at the indicated E:T ratio with MCF-7 or MDA-MB-231 cells (target cells) pretreated with anti-PD-L1 (10 μg/mL) or not for 6 hours. Dead target cells out of the total target cells were determined.

Supplementary Figure 6 1-MT treatment alone did not induce tumor cell apoptosis. **(A)** IDO1 inhibitor Lindrostat facilitated the cytotoxicity of Vγ9Vδ2 T cells against MDA-MB-231 cells, but not MCF-7 cells. MCF-7 or MDA-MB-231 cells (target) were co-cultured with Vγ9Vδ2 T cells (effector) with Lindrostat or vehicle for 6 hours. The percentage of dead cells out of total target cells was shown. *n*=3. **(B, C)** MCF-7 and MDA-MB-231 cells were treated with 1-MT (500 μM), Lindrostat (10, 100, 1000 nM) or vehicle for 6 or 12 hours. Apoptotic cells (PI^+^) were detected by flow cytometry. The data were representative of three independent experiments. Data represented mean ± SD; unpaired Student’s *t*-test. Significance was set to *P* < 0.05 and represented as **P* < 0.05, ***P* < 0.01, ****P* < 0.001, and *****P <* 0.0001, *n.s.*, not significant.

Supplementary Figure 7 1-MT treatment did not promote NKG2D expression of Vγ9Vδ2 T cells stimulated with MDA-MB-231 cells or MCF-7 cells. Human Vγ9Vδ2 T cells co-cultured with MCF-7 or MDA-MB-231 cells were treated with 1-MT (500 μM) or vehicle for 6 hours. Expression of NKG2D on Vγ9Vδ2 T cells from healthy donors was shown (*n*=3). Data represented mean ± SD; unpaired Student’s *t*-test. Significance was set to *P* < 0.05 and represented as **P* < 0.05, ***P* < 0.01, ****P* < 0.001, and *****P <* 0.0001, *n.s.*, not significant.

Supplementary Figure 8 MICA/B levels on MDA-MB-231 and MCF-7 cells. Representative histograms of MICA/B expression by MCF-7 (luminal A) and MDA-MB-231 (TNBC) cells.
