# Supplementary figures and images for: IDO Inhibition Facilitates Antitumor Immunity of Vγ9Vδ2 T Cells in Triple-Negative Breast Cancer
Source: Front Oncol. 2021 Jul 22;11:679517. doi: 10.3389/fonc.2021.679517 (PMC8351331; doi:10.3389/fonc.2021.679517)

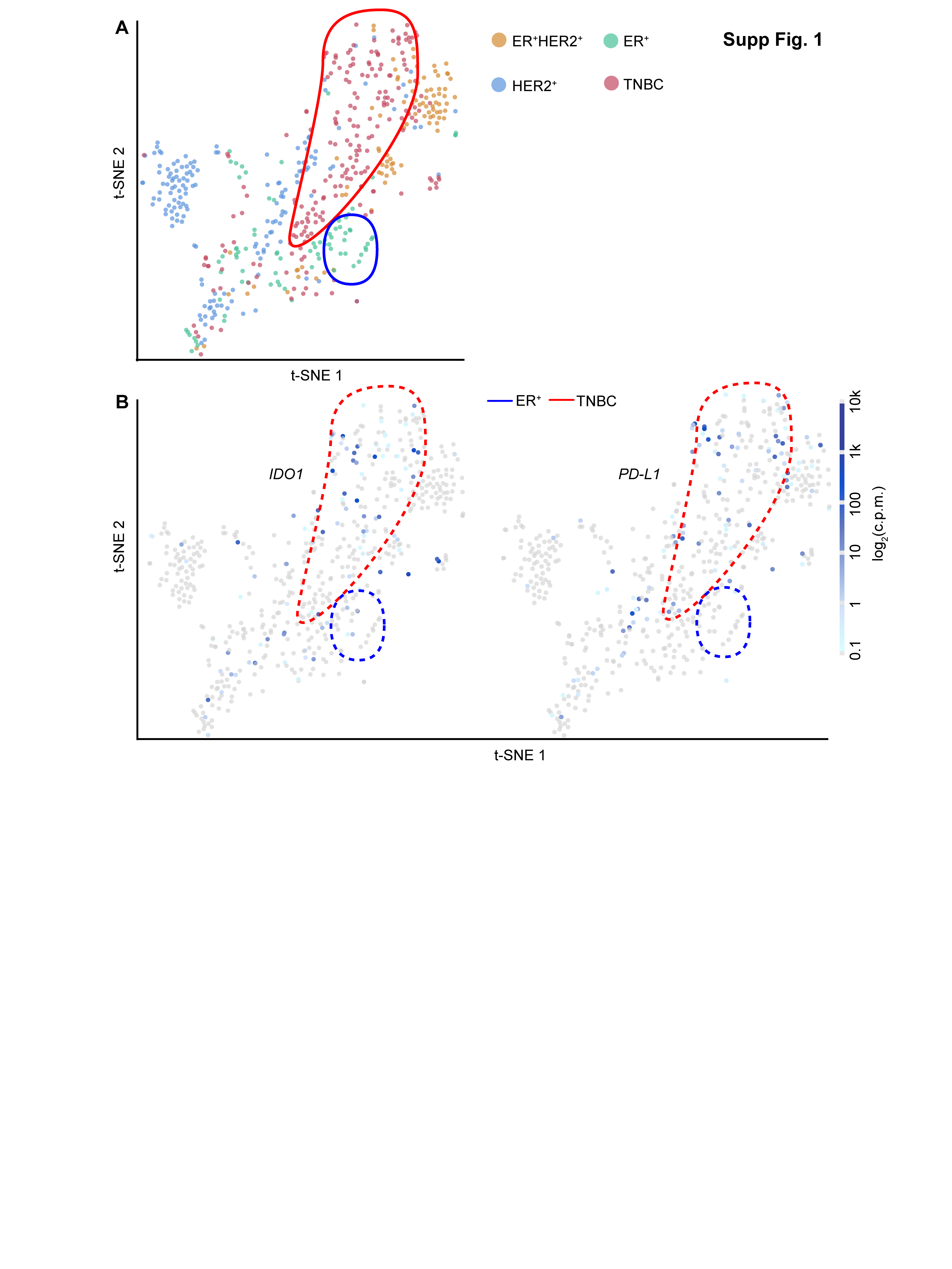

Supplement: Supplementary Figure 1 — The levels of PD-L1 and IDO1 in TNBC and Luminal A breast cancers. (A) t-SNE plot of all 515 classified cells, demonstrating separation by cell type. Individual cells were colored green for luminal A, yellow for luminal B, blue for HER2, and red for TNBC tumors (The Single Cell Expression Atlas data base). (B) Expression levels of IDO1 and PD-L1 across 515 single cells illustrated in t-SNE plots. Individual cells were colored red for TNBC, blue for luminal A (The Single Cell Expression Atlas data base). [file Image_1.tif]

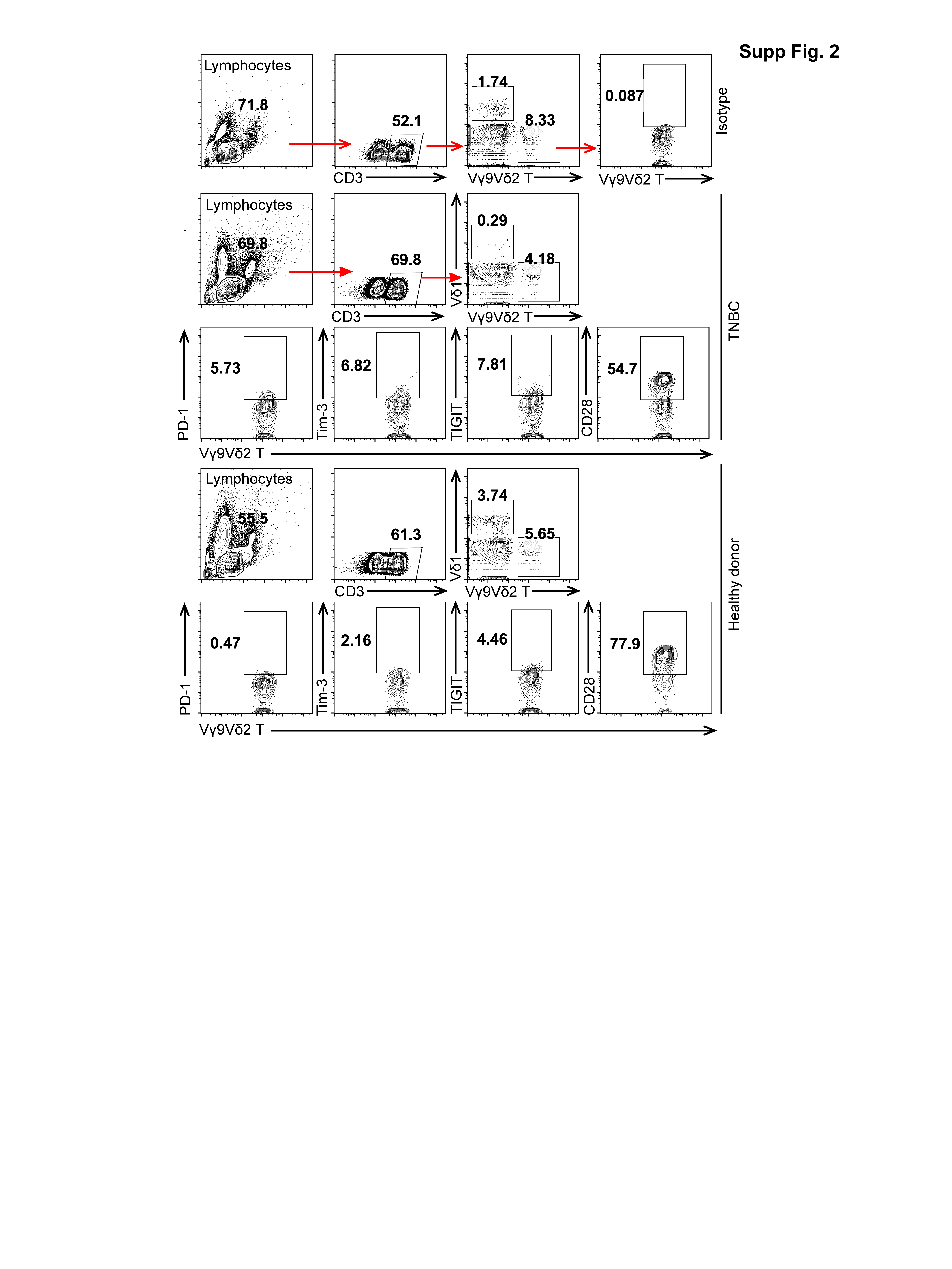

Supplement: Supplementary Figure 2 — Identification of exhaustion and costimulatory markers in triple negative breast cancer patients. PD-1+, Tim-3+, TIGIT+, and CD28+ levels on Vγ9Vδ2+ T cells in PBMCs of healthy donors and triple negative breast cancer patients (TNBC) were shown. [file Image_2.tif]

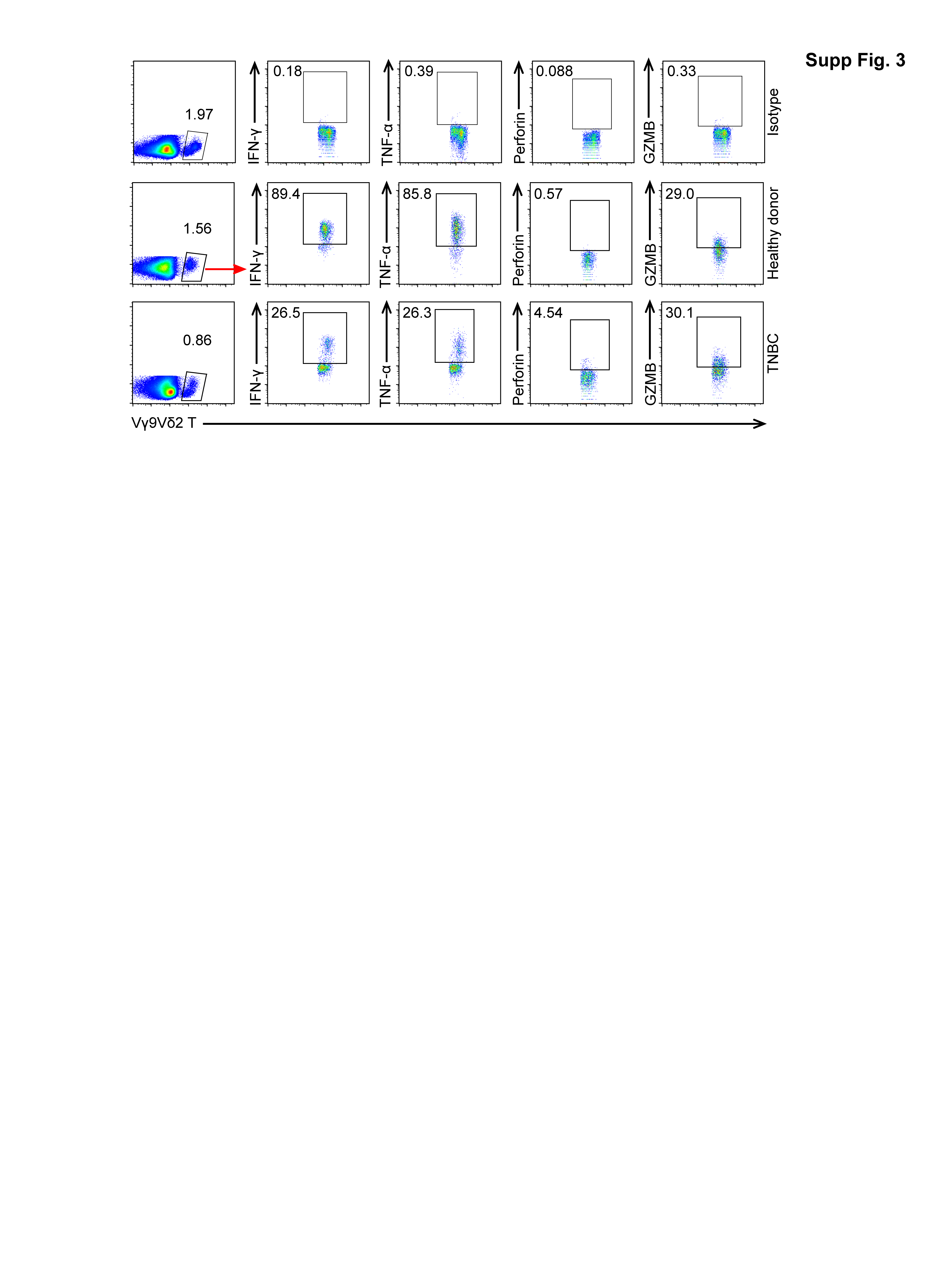

Supplement: Supplementary Figure 3 — Cytokine production of Vγ9Vδ2+ T cells. Frequency of TNF-α+, IFN-γ+, Perforin+, and Granzyme B+ Vγ9Vδ2+ T cells in healthy donor and triple negative breast cancer samples were shown. [file Image_3.tif]

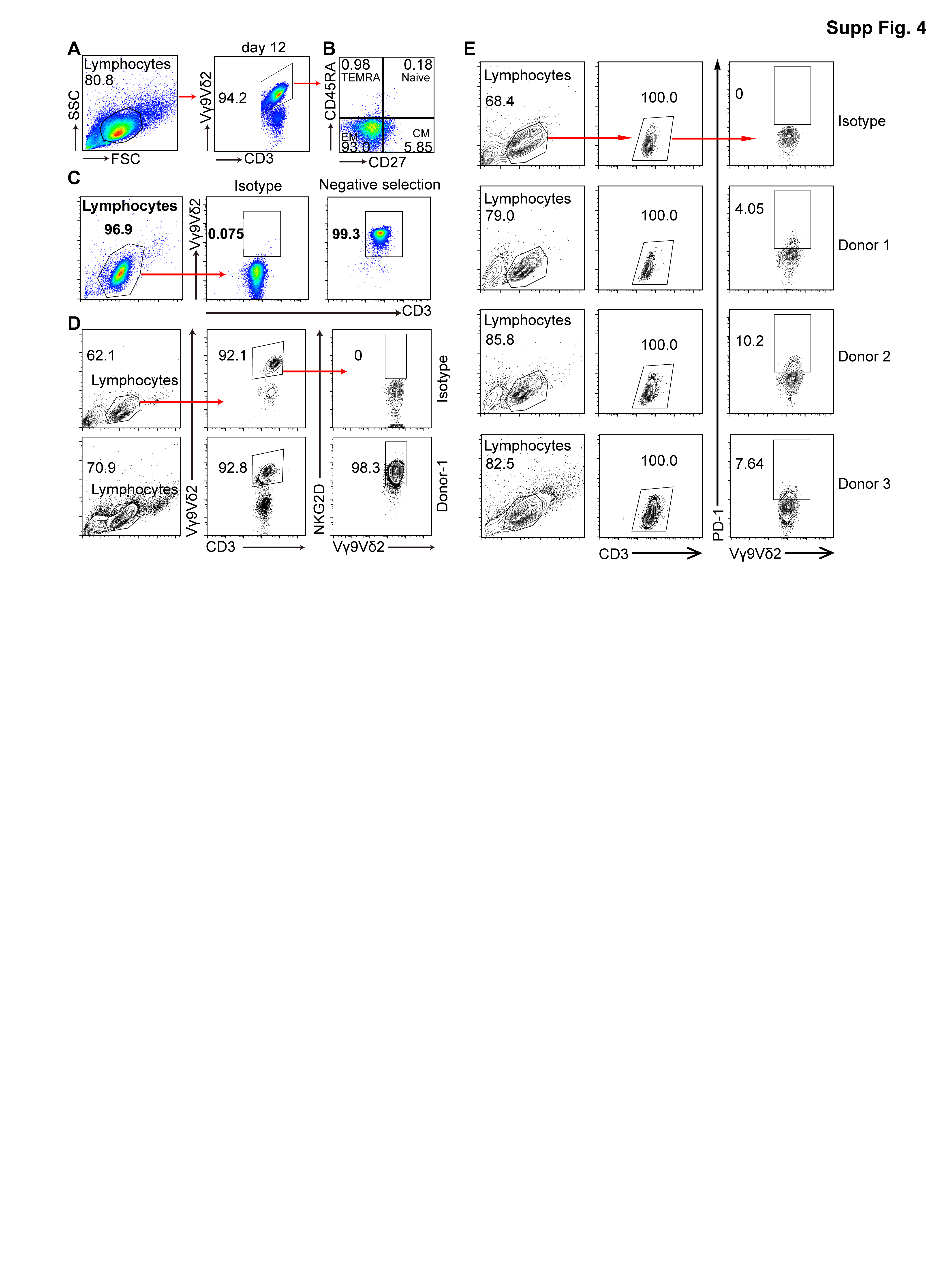

Supplement: Supplementary Figure 4 — Percentage of NKG2D+, PD-1+ Vγ9Vδ2 T cells out of the total Vγ9Vδ2 T lymphocyte population. (A, B) Representative flow cytometry plots showing the gating strategy to identify lymphocytes including subsets of Vγ9Vδ2 T cells expanded from ZOL. (C) Vγ9Vδ2 T cells were further purified by negative selection with EasySep™ Human Gamma/Delta T Cell Isolation Kit. (D, E) Vγ9Vδ2 T cells were expanded in vitro from the human peripheral blood cells with ZOL. Frequency of NKG2D and PD-1 expression on Vγ9Vδ2+ T cells at day 12 was shown. [file Image_4.tif]

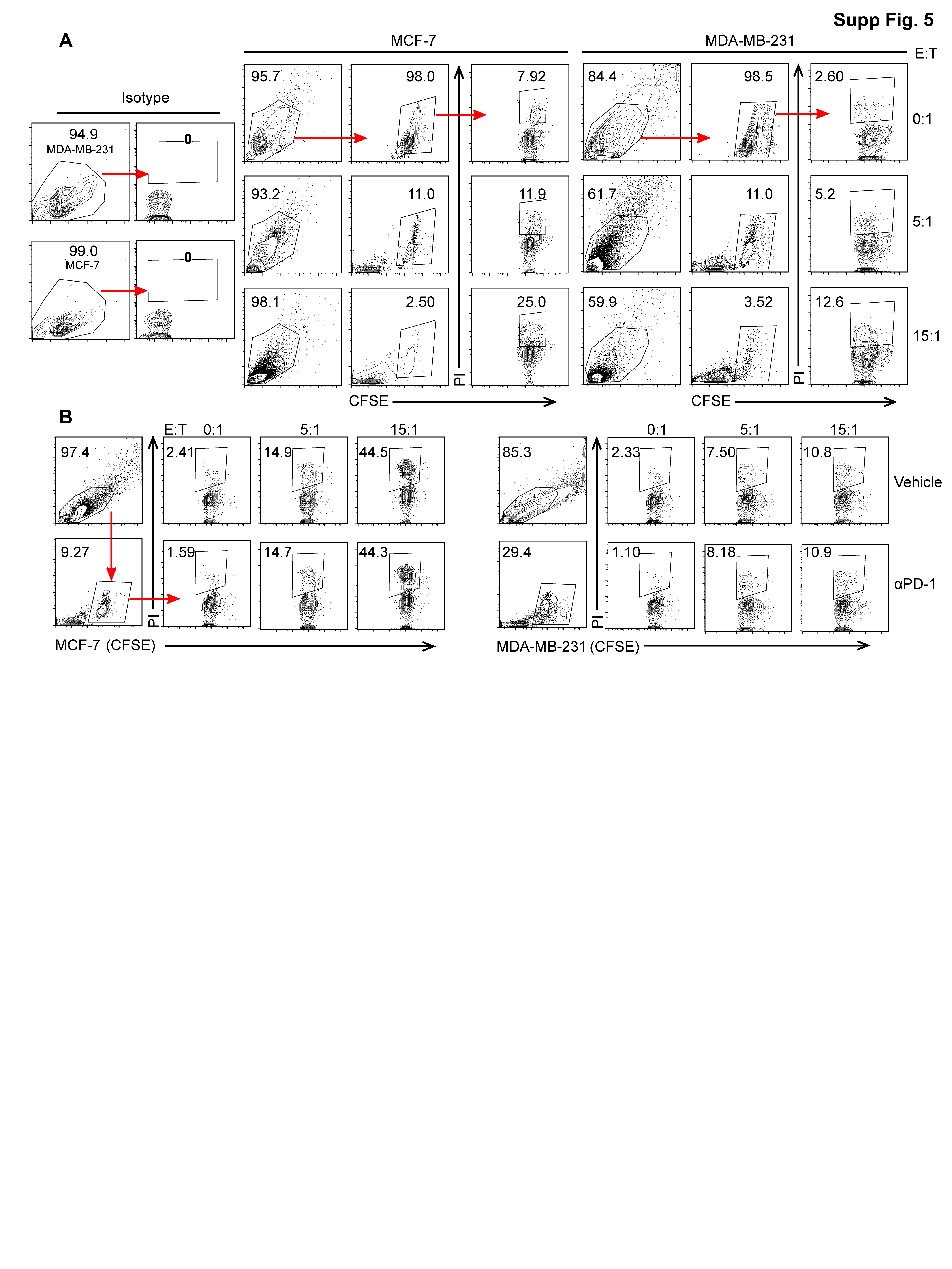

Supplement: Supplementary Figure 5 — Anti-PD-L1 antibody could not further enhance the antitumor efficacy of Vγ9Vδ2 T cells. (A) Cytotoxicity of Vγ9Vδ2 T cells toward MCF-7 or MDA-MB-231 cell lines at the indicated ratio of effector to target (E:T). Frequency of dead cells out of whole target cells were showed as PI+. (B) Cytotoxicity of Vγ9Vδ2 T cells had no obvious difference at the indicated E:T ratio with MCF-7 or MDA-MB-231 cells (target cells) pretreated with anti-PD-L1 (10 μg/mL) or not for 6 hours. Dead target cells out of the total target cells were determined. [file Image_5.tif]

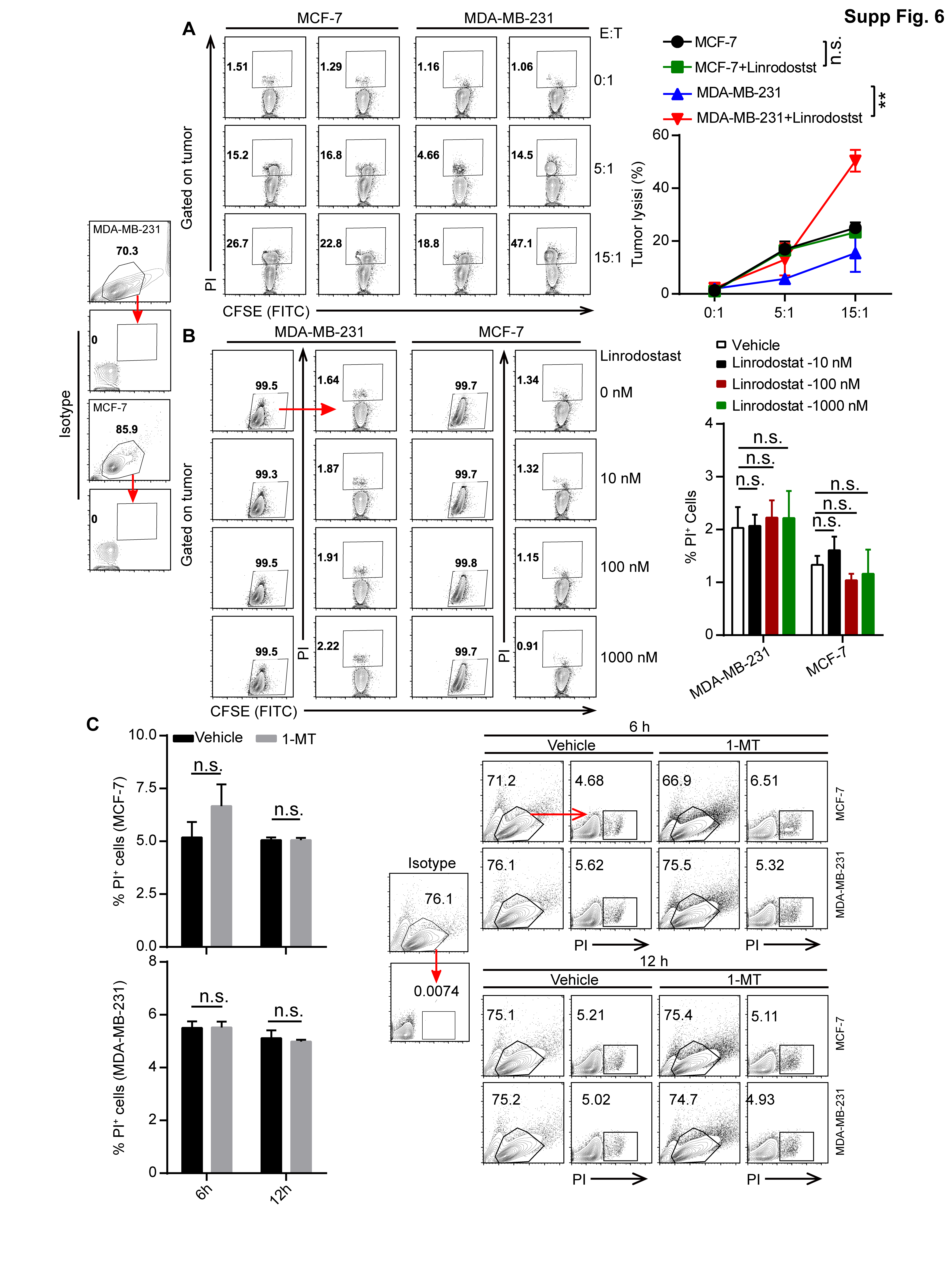

Supplement: Supplementary Figure 6 — 1-MT treatment alone did not induce tumor cell apoptosis. (A) IDO1 inhibitor Lindrostat facilitated the cytotoxicity of Vγ9Vδ2 T cells against MDA-MB-231 cells, but not MCF-7 cells. MCF-7 or MDA-MB-231 cells (target) were co-cultured with Vγ9Vδ2 T cells (effector) with Lindrostat or vehicle for 6 hours. The percentage of dead cells out of total target cells was shown. n=3. (B, C) MCF-7 and MDA-MB-231 cells were treated with 1-MT (500 μM), Lindrostat (10, 100, 1000 nM) or vehicle for 6 or 12 hours. Apoptotic cells (PI+) were detected by flow cytometry. The data were representative of three independent experiments. Data represented mean ± SD; unpaired Student’s t-test. Significance was set to P < 0.05 and represented as *P < 0.05, **P < 0.01, ***P < 0.001, and ****P < 0.0001, n.s., not significant. [file Image_6.tif]

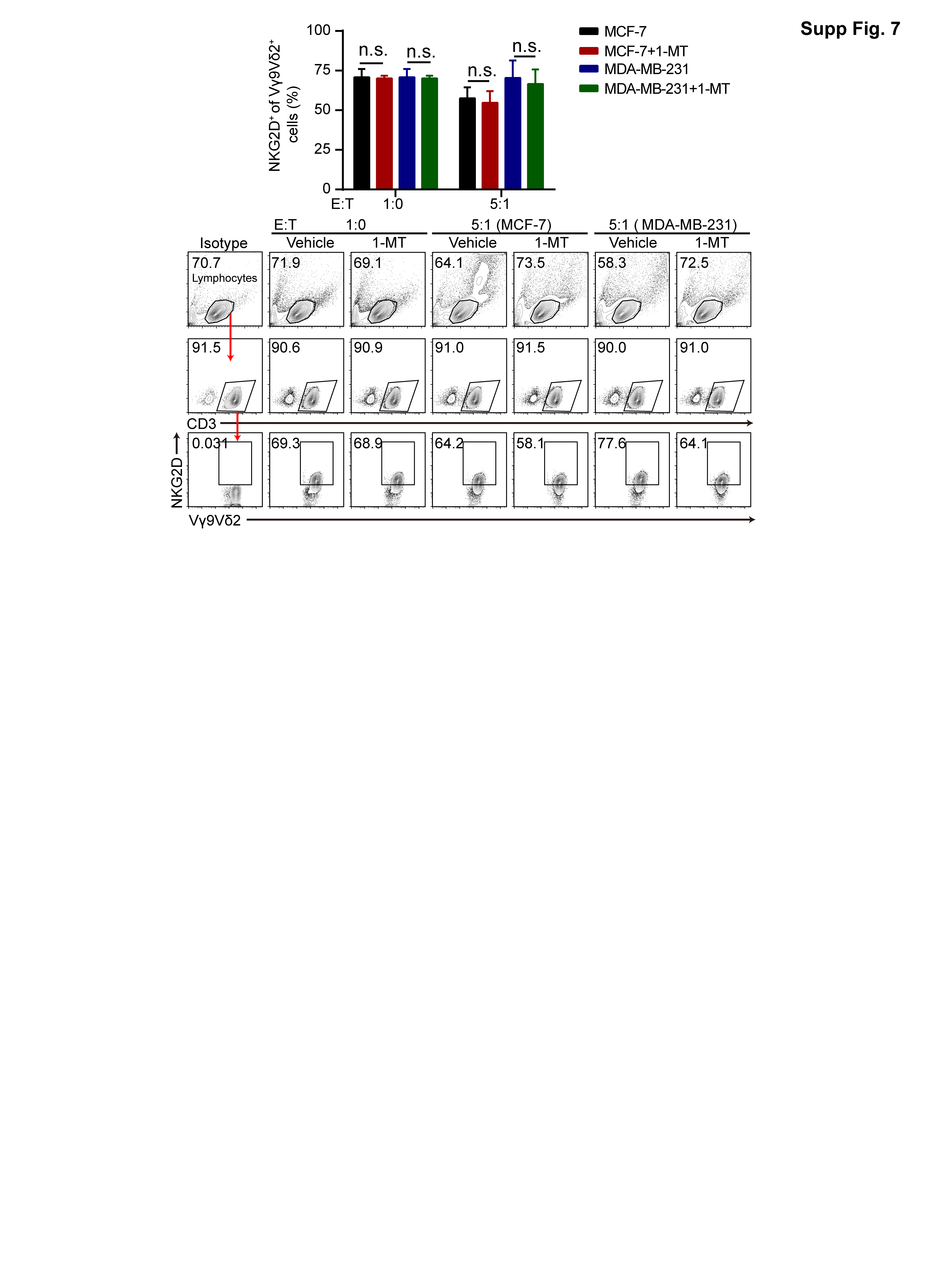

Supplement: Supplementary Figure 7 — 1-MT treatment did not promote NKG2D expression of Vγ9Vδ2 T cells stimulated with MDA-MB-231 cells or MCF-7 cells. Human Vγ9Vδ2 T cells co-cultured with MCF-7 or MDA-MB-231 cells were treated with 1-MT (500 μM) or vehicle for 6 hours. Expression of NKG2D on Vγ9Vδ2 T cells from healthy donors was shown (n=3). Data represented mean ± SD; unpaired Student’s t-test. Significance was set to P < 0.05 and represented as *P < 0.05, **P < 0.01, ***P < 0.001, and ****P < 0.0001, n.s., not significant. [file Image_7.tif]

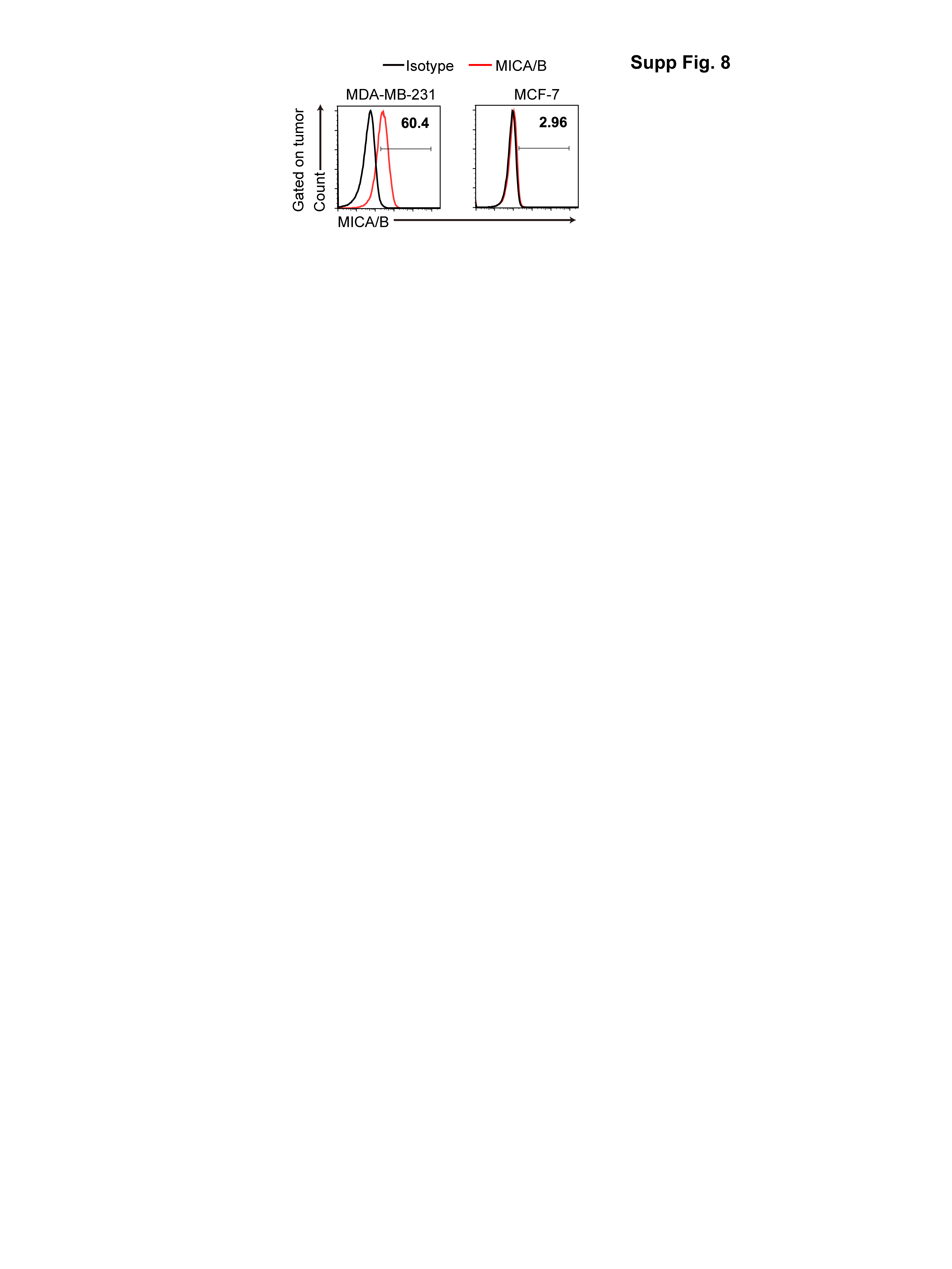

Supplement: Supplementary Figure 8 — MICA/B levels on MDA-MB-231 and MCF-7 cells. Representative histograms of MICA/B expression by MCF-7 (luminal A) and MDA-MB-231 (TNBC) cells. [file Image_8.tif]
